# Hierarchical Data Fusion Algorithm for Multiple Wind Speed Sensors in Anemometer Tower

**DOI:** 10.3390/s26020565

**Published:** 2026-01-14

**Authors:** Junhong Duan, Hailong Zhang, Chao Tu, Jun Song, Wei Niu, Zhen Zhang, Jinze Han, Jiuyuan Huo

**Affiliations:** 1 Gansu Electric Power Company of State Grid, Lanzhou 730046, China; duanjh@gs.sgcc.com.cn (J.D.); 90002607@gs.sgcc.com.cn (H.Z.); cnsongjun@gs.sgcc.com (J.S.); cnniuw@gs.sgcc.com.cn (W.N.); 2 Zhangye Power Supply Company of Gansu Electric Power Company of State Grid, Zhangye 734000, China; gansutuchao@126.com (C.T.); 18893105073@163.com (Z.Z.); 18993665252@163.com (J.H.); 3 School of Electronic and Information Engineering, Lanzhou Jiaotong University, Lanzhou 730070, China

**Keywords:** aquila optimizer, data fusion, extreme learning machine (ELM), Q-learning, unscented kalman filter (UKF)

## Abstract

Accurate and reliable wind speed measurement is essential for applications such as wind power generation and meteorological monitoring. Data fusion from multiple anemometers mounted on wind measurement towers is a key approach to obtaining high-precision wind speed information. In this study, a hierarchical data fusion strategy is proposed to enhance both the quality and efficiency of multi-sensor fusion on wind measurement towers. At the local fusion stage, multi-sensor wind speed data are denoised and fused using an unscented Kalman filter enhanced with fuzzy logic and a robustness factor (FLR-UKF). At the global decision fusion stage, decision-level fusion is achieved through an extreme learning machine (ELM) neural network optimized by a Q-learning-improved Aquila optimizer (QLIAO-ELM). By incorporating a spiral surrounding attack mechanism and a Q-learning-based adaptive strategy, QLIAO-ELM significantly enhances global search capability and convergence speed, enabling the ELM network to obtain superior parameters within limited computational time. Consequently, the accuracy and efficiency of decision fusion are improved. Experimental results show that, during the local fusion phase, the RMSE of FLR-UKF is reduced by 26.46% to 28.6% compared to the traditional UKF; during the global fusion phase, the RMSE of QLIAO-ELM is reduced by 27.1% and 14.0% compared to ELM and ISSA-ELM, respectively.

## 1. Introduction

With the growing global demand for clean energy and the advancement of meteorological research, wind speed has emerged as a critical parameter whose accurate measurement is essential for the efficient operation of wind farms, the reliability of weather forecasting, and the progress of related scientific studies [1,2]. Wind measurement towers, as important facilities for acquiring wind speed data, are typically equipped with multiple anemometers installed at different heights and orientations to capture wind information from various perspectives [3]. However, in practical applications, anemometers are highly susceptible to complex environmental factors such as strong winds, dust, temperature fluctuations, and electromagnetic interference. As a result, the data collected by a single sensor are often affected by noise, bias, or occasional faults, which compromises both accuracy and reliability, making it difficult to satisfy the requirements of high-precision applications [4]. Consequently, the effective fusion of multi-sensor wind speed data to enhance measurement accuracy and stability has become a significant challenge and a focal point of current research.

Multi-sensor data fusion, as an effective data processing approach, integrates redundant and complementary information provided by multiple sensors to obtain more comprehensive and accurate target information than that derived from a single sensor [5,6,7]. In the field of wind speed measurement, such techniques can significantly improve data quality by reducing the influence of noise and outliers, thereby enhancing system reliability and robustness [8]. Current data fusion methods include simple averaging, weighted averaging, Kalman filtering and its variants, particle filtering, and machine learning-based fusion algorithms [9,10]. Although simple and weighted averaging methods are easy to implement, their fusion performance is poor when handling noisy data with outliers, as they fail to fully exploit the underlying information. Kalman filtering and its improved variants perform well in linear dynamic systems; however, their effectiveness is limited when applied to nonlinear systems or wind speed data in complex environments [11]. Particle filtering can better address nonlinear and non-Gaussian noise problems, but its high computational complexity restricts real-time applications [12]. Machine learning-based methods, such as artificial neural networks and support vector machines, possess strong learning and generalization capabilities and can improve fusion accuracy to some extent. Nevertheless, these algorithms typically require large amounts of training data [13,14] Moreover, sensor noise and uncertainty directly affect fusion results, and the robustness of machine learning models remains a critical challenge when facing outliers or missing data. Without addressing the quality issues of data sources, even the most advanced machine learning algorithms may fail to achieve the expected performance.

To address the limitations of existing data fusion methods, this paper proposes a hierarchical fusion algorithm for multi-anemometer wind measurement towers. The algorithm adopts a layered architecture, dividing the fusion process into primary data fusion and secondary decision fusion stages. In the primary data fusion stage, an improved unscented Kalman filter (UKF) is employed to process the data collected from multiple wind speed sensors. The UKF, based on the unscented transformation, generates sigma points through deterministic sampling, enabling a more accurate approximation of the probability distribution of nonlinear functions. This effectively overcomes the errors introduced by linearization in traditional Kalman filtering when dealing with nonlinear systems. Furthermore, by incorporating a fuzzy logic-based adaptive weighting mechanism to dynamically adjust the noise covariance weights and introducing a robustness factor to suppress outliers, the fused results more accurately reflect the true wind speed conditions.

In the secondary decision fusion stage, an improved Aquila Optimizer (AO) [15] is introduced to optimize the Extreme Learning Machine (ELM) neural network [16]. AO is a recently developed intelligent optimization algorithm inspired by the unique predatory behavior of eagles, and it demonstrates strong global search capability and fast convergence. To further enhance its performance, the spiral encircling strategy and Q-learning mechanism [17] are incorporated, thereby improving the balance between global exploration and local exploitation and accelerating convergence. The improved AO is employed to optimize the input weights and biases of the ELM network, resulting in the QLIAO-ELM model. This model is capable of significantly improving the efficiency of decision-level fusion while maintaining the required accuracy, thus meeting the demands of real-time data fusion.

The hierarchical fusion algorithm proposed in this study integrates the noise-reduction capability of the improved UKF with the efficiency of the QLIAO-ELM neural network in decision-level fusion. The approach is designed to provide a more accurate, reliable, and real-time solution for multi-sensor wind speed data fusion in meteorological towers. Compared with conventional fusion methods, the proposed hierarchical strategy not only effectively suppresses noise in wind speed sensor data but also ensures real-time processing while maintaining high measurement accuracy.

The main contributions and works of this paper are as follows:

(1) A fuzzy logic-based adaptive weighting mechanism combined with an anti-interference unscented Kalman filter (FLR-UKF) is proposed and applied to the local fusion of multi-anemometer data. Since fuzzy logic does not require the assumption of Gaussian noise distribution, it is more suitable for nonlinear system filtering in complex environments such as wind measurement towers, thereby effectively improving the accuracy of sensor data.

(2) An AO algorithm based on the spiral encircling attack strategy [18] and a Q-learning mechanism (QLIAO) is designed, and QLIAO is employed to optimize the ELM for global decision-level data fusion. In this process, the output of local fusion is used as the input for global fusion, which not only reduces data redundancy but also enhances the accuracy of wind speed data fusion.

(3) Through experimental validation, the superiority of the proposed hierarchical data fusion strategy in multi-anemometer data fusion is demonstrated, and comparisons with conventional methods are conducted. The results indicate that the proposed approach achieves significant advantages in terms of fusion accuracy.

The remainder of this paper is organized as follows. Section 2 presents a review of related work. Section 3 introduces the proposed data fusion framework. Section 4 details the design of the fusion algorithm, including the improved UKF for local fusion and the global decision-level fusion algorithm. Section 5 provides simulation validation and performance analysis. Finally, Section 6 concludes the paper and outlines directions for future research.

## 2. Related Work

Multi sensor data fusion (MDF) techniques effectively overcome the limitations of individual sensors in measurement range, accuracy, and robustness by collaboratively processing distributed sensing data. Through a multi-level data processing workflow—including data acquisition, preprocessing, and information estimation—the resulting fused information provides critical support for control system decision-making. MDF has been widely applied in environmental monitoring [7,19], civil infrastructure monitoring [20,21], and industrial monitoring [4,22].

The co-design of efficient fusion algorithms and adaptable frameworks remains a core challenge for enhancing the performance of multi sensor systems. Cao et al. [23] proposed a mobile heterogeneous wireless sensor network (WSN) data fusion algorithm based on a bat algorithm-optimized extreme learning machine (BAT-ELM). In this approach, sensor nodes are treated as neurons in an ELM neural network, and the bat algorithm is employed to optimize the randomly initialized network weights and biases, significantly improving fusion stability and efficiency. Tabella et al. [24] investigated the application of WSNs in oil spill detection and localization for underwater production systems. Passive acoustic sensors deployed on subsea templates detect energy signals, enabling nodes to autonomously determine the presence of oil, while a fusion center (FC) aggregates local decisions to form a global judgment.

Webber and Rojas [25] conducted a systematic comparison of sensor-level, feature-level, and decision-level data fusion strategies for human activity recognition (HAR) using multiple sensors. Evaluations on four public datasets and four machine learning classifiers revealed that decision-level fusion achieved the highest accuracy, albeit at the cost of increased computational time. In contrast, Kalman filtering at the feature level demonstrated the best balance, combining high accuracy with minimal processing latency, making it more suitable for real-time applications in wearable devices. These findings provide a valuable benchmark for data fusion strategies in the HAR domain.

Yunas and Ozanyan [26] further emphasized that the superiority of the fusion algorithm has a far greater impact on result accuracy than the inherent performance of the sensors themselves, highlighting the critical importance of algorithmic innovation.

The performance of collaborative multi sensor fusion generally surpasses that of individual sensors [27,28], and this advantage is particularly evident in hybrid frameworks combining filtering techniques and neural networks. Xia et al. [29] addressed the challenges of uneven temperature distribution and low data acquisition efficiency in WSNs deployed in smart greenhouses by proposing a hierarchical real-time fusion strategy. In this WSN, a three-tier architecture was adopted: the bottom layer consisted of sensors that acquired and preprocessed temperature data using an improved UKF; intermediate cluster-head nodes served as local fusion centers, applying a parallel inverse covariance intersection (ICI) algorithm to fuse bottom-layer data; the top layer global fusion center integrated intermediate-layer data using an enhanced ELM algorithm to achieve real-time representation of greenhouse-wide temperature. Simulations demonstrated that this strategy significantly improved fusion accuracy and real-time performance, thereby optimizing the overall temperature monitoring system.

Yang et al. [30] introduced a decaying memory matrix and sequential analysis fusion estimator to improve UKF, resulting in the sequential analysis and inverse covariance intersection-global state fusion estimation (SICI-GSFE) algorithm. This approach achieved the fusion of 600 temperature datasets within 1.972 s, demonstrating a 2.41-fold improvement in efficiency compared with batch fusion methods.

For meteorological parameter fusion, Shi et al. [31] proposed a parallel deep predictive model (PDCI) that fuses multiple time-series datasets using a covariance intersection algorithm. In humidity monitoring, this method reduced RMSE and MAE by 7.08% and 0.55% compared with the STL-GRU (Seasonal trend decomposition using loess and gated recurrent unit) model, respectively, although its conservativeness and consistency were inferior to the standard ICI method. In the field of wind speed prediction, Chen et al. [32] developed the VMD-ISOA-KELM model (Variational modal decomposition-improved seagull optimization algorithm-kernel extreme learning machine), in which an improved seagull optimization algorithm (ISOA) was used to optimize kernel ELM parameters. The model achieved MAE = 0.182 and RMSE = 0.235 in short-term wind speed prediction, reducing errors by 27.5% and 25.6% compared with VMD-KELM. However, early convergence issues remained. Subsequently, the LSOA (Improvement seagull optimization algorithm based on levy flight) algorithm [33] employed a Levy flight strategy to enhance global search ability, shortening the optimal path length by 7.5%, but at the cost of a 5.5-fold increase in computation time, revealing the trade-off between accuracy and efficiency.

In this study, a hierarchical sensor data fusion framework is adopted. At the bottom layer, sensor data are grouped and locally fused using the FLR-UKF, improving the accuracy of low-level data and providing a foundation for global fusion. During the global fusion process, the outputs from local fusion serve as inputs to the global fusion stage, where the QLIAO-ELM model ensures precise and rapid processing of wind speed data. This framework provides an effective algorithmic solution for real-time and accurate wind speed monitoring.

## 3. Data Fusion Strategy

### 3.1. Data Fusion Model

**Figure 1:**
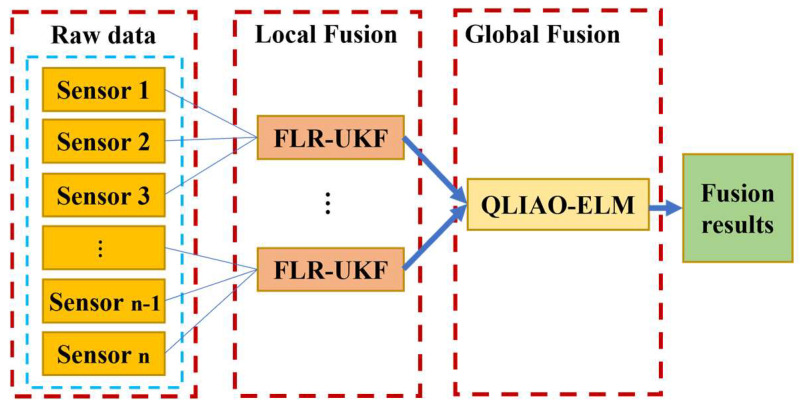
Hierarchical Data Fusion Framework.

To address the requirements of multi-wind-speed sensor data fusion at meteorological towers, this study proposes a hierarchical multi-sensor data fusion model, as illustrated in Figure 1. The model is organized into three primary layers: the raw data layer, the local fusion layer, and the global fusion layer. At the raw data layer, individual wind speed sensors collect original wind speed measurements. In the local fusion layer, the raw data are first grouped and then processed through multiple FLR-UKF modules to perform preliminary integration of local information. The locally fused outputs are subsequently transmitted to the global fusion layer, where the QLIAO-ELM module conducts global-level fusion computations. The final output represents the integrated wind speed data, enabling accurate and reliable multi-sensor wind speed fusion.

### 3.2. Proposed FLR-UKF for Local Fusion

The UKF [34,35], effective in addressing state estimation problems for nonlinear dynamic systems, is highly dependent on the prior specification of process and observation noise statistics. In enclosed or structured industrial environments, noise parameters can typically be obtained through offline calibration, rendering the UKF relatively stable. However, in meteorological tower environments, disturbances such as severe weather, strong wind impacts, and equipment vibrations often occur, leading to noise characteristics that are significantly non-stationary and non-Gaussian, with prior information being difficult to model accurately. Under fixed noise covariance assumptions, the filter is prone to state drift or even numerical divergence. To overcome this limitation, a novel algorithm is proposed in this study, namely FLR-UKF. In FLR-UKF, fuzzy inference is employed to dynamically adjust the weighting coefficients of noise covariance, thereby eliminating the need to assume prior probability distributions of noise. This makes the method more suitable for nonlinear system filtering in complex environments such as meteorological towers. In addition, a robustness factor is incorporated to suppress outliers, while a triangular decomposition matrix is applied to resolve non-positive-definiteness issues, resulting in an adaptive filtering algorithm with enhanced robustness.

#### 3.2.1. Local State Estimation

The wind speed state of the meteorological tower is described by the following discrete-time state-space model as (1) [36,37,38].

(1) $$X_{g+1}=A_{g}X_{g}+\omega_{g},g=0,1,\cdots ,$$

where $X_{g+1}$ is the wind speed state at the next time g+1. $A_{g}$ is the system matrix, and $\omega_{g}$ is the noise, which is typically assumed to be zero-mean Gaussian white noise with a nonnegative definite covariance matrix $Q_{g}$. The measurement equation for each sensor is provided as (2).

(2) $$Z_{\left(r,g\right)}=H_{\left(r,g\right)}X_{g}+V_{\left(r,g\right)},r=0,1,$$

where *Z*_(*r*,*g*)_ is the wind speed measured by the *r*-th sensor at the time g, where *H*_(*r*,*g*)_ denotes the output matrix and *V*_(*r*,*g*)_ represents the observation noise of the *r*-th sensor. The latter is generally assumed to be zero-mean Gaussian white noise.

For the collected raw data, an improved UKF is applied to perform local fusion processing. A detailed description of the improved UKF is provided in the following section.

#### 3.2.2. Fuzzy Logic-Based Adaptive Weighting Mechanism

The inputs of the fuzzy system are defined as the normalized value of the innovation sequence ${\tilde{z^{\prime }}}_{g}$ and the innovation covariance ratio $r_{g}$.

(3) $${\tilde{z^{\prime }}}_{g}=\frac{∥{\tilde{z}}_{g}∥}{\sqrt{tr(P_{zg})}},$$

where ${\tilde{z}}_{g}=z_{g}-{\hat{z}}_{g}$ denotes the innovation, and directly reflects the inconsistency between the system’s predictions and actual observations. However, the raw innovation is influenced by dimensionality and numerical range, making it difficult to be directly used for rule judgment in fuzzy logic. Therefore, we define the normalized innovation ${\tilde{z^{\prime }}}_{g}$ where $∥{\tilde{z}}_{g}∥$ denotes the norm of the innovation vector. $tr(P_{zg})$ represents the trace of the innovation covariance matrix, which reflects the overall fluctuation range of the prediction error (the subscript *z* corresponds to the innovation variable).

(4) $$r_{g}=\frac{tr(C_{0,g})}{tr(P_{zg})},$$

where $C_{0,g}$ denotes the sliding innovation covariance.

**Figure 2:**
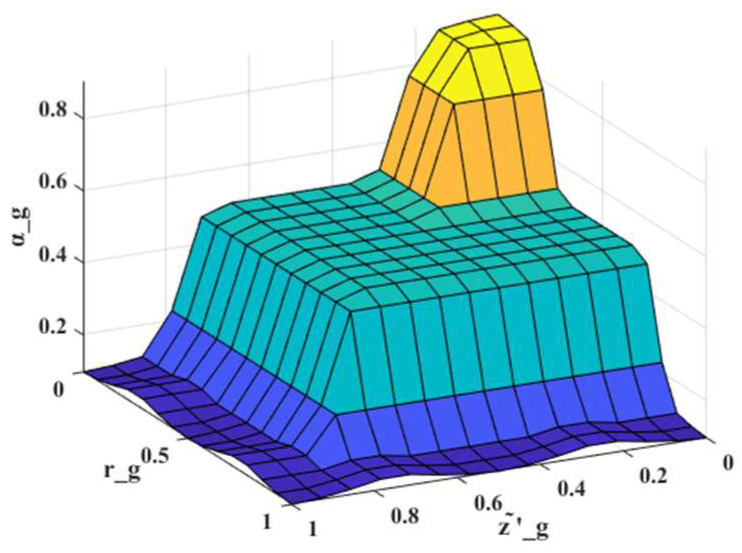
Visualization of the fuzzy rules.

The normalized innovation ${\tilde{z^{\prime }}}_{g}$ and the covariance ratio $r_{g}$ are defined as the inputs of the fuzzy system, while the weighting coefficient $\alpha_{g}$ is generated as the output and determined through centroid defuzzification [21]. Figure 2 shows the impact of the normalized innovation ${\tilde{z^{\prime }}}_{g}$ and the covariance ratio $r_{g}$ on the weighting coefficient $\alpha_{g}$. This figure is generated based on the fuzzy rules presented in this paper. The fuzzy rules are designed according to the following principle: when both ${\tilde{z^{\prime }}}_{g}$ and $r_{g}$ are relatively small, the system state is considered stable, and a larger value of $\alpha_{g}$ assigned, allowing the algorithm to rely more heavily on the prior model. Conversely, when ${\tilde{z^{\prime }}}_{g}$ and $r_{g}$ are large, abnormal residuals or biased covariance are indicated, and a smaller value of $\alpha_{g}$ adopted, enabling the algorithm to depend more on real-time measurement updates. To ensure smooth transitions in the fuzzy reasoning process, triangular membership functions are employed for both inputs and outputs.

The system and measurement noise covariances are adjusted as defined in (5) and (6), respectively.

(5) $$Q_{g}=\alpha_{g}\cdot Q_{g-1}+(1-\alpha_{g})\cdot Q_{cmp},$$

(6) $$R_{g}=\left(\begin{array}{c} 1-\alpha_{g} \end{array}\right)\cdot R_{g-1}+\alpha_{g}\cdot R_{cmp},$$

where *Q_cmp_* and *R_cmp_* denote the empirical process and measurement noise covariances, respectively.

#### 3.2.3. Robustness Factor Correction

The innovation covariance is corrected using the robustness factor $c_{g}$, which is defined in (7). The corrected innovation covariance is given by (8).

(7) $$c_{g}=\max\{1,\frac{tr(C_{0,g})}{tr(P_{zg})}\},$$

(8) $$P_{zg}^{\prime }=c_{g}\cdot P_{zg},$$

#### 3.2.4. Triangular (LU) Decomposition

To avoid the issue of non-positive definiteness, the error covariance P is decomposed using *LU* decomposition, as expressed in (9). Subsequently, the sigma points are adjusted accordingly.

(9) $$P=L\cdot U\Rightarrow \sqrt{P}=L\cdot \sqrt{U},$$

where P is the wind speed state error covariance matrix, L is an orthogonal matrix, and U is a diagonal matrix. An orthogonal matrix L satisfies $L^{T}=L^{-1}$, and the square root $\sqrt{U}$ of a diagonal matrix U remains a diagonal matrix.

In this paper, L is a special orthogonal matrix adapted to the covariance matrix, which satisfies idempotency ($L^{2}=L$) and commutes with the diagonal matrix $\sqrt{U}$. The covariance matrix P is a symmetric positive semi-definite matrix, and its specialized LU decomposition (with L orthogonal and U diagonal) is essentially equivalent to a simplified form of spectral decomposition. At this point, $\sqrt{P}=L\sqrt{U}$ is a valid derivation of the matrix square root, which not only solves the collapse problem of the standard UKF caused by non-positive definiteness in Cholesky decomposition but also meets the algorithm’s requirements for numerical stability and filtering robustness.

The overall procedure of the FLR-UKF is summarized as follows:

Step 1: Initialization of the state and covariance.

(10) $${\hat{x}}_{0}=E[x_{0}],$$

(11) $$P_{0}=E[(x_{0}-{\hat{x}}_{0}){\left(x_{0}-{\hat{x}}_{0}\right)}^{T}],$$

Step 2: Generation of sigma points and corresponding weights via *LU* decomposition:

(12) $$\left\{\begin{array}{l} \chi_{g-1}^{\left(0\right)}={\hat{x}}_{g-1}\\ \chi_{g-1}^{\left(s\right)}={\hat{x}}_{g-1}+{\left(\sqrt{n+\lambda }\cdot L\cdot \sqrt{U_{\left(i\right)}}\right)}_{s},s=1,\ldots ,n\\ \chi_{g-1}^{\left(s+n\right)}={\hat{x}}_{g-1}-{\left(\sqrt{n+\lambda }\cdot L\cdot \sqrt{U_{\left(i\right)}}\right)}_{s},s=1,\ldots ,n\\ W_{m}^{\left(0\right)}=W_{c}^{s}=W_{s+n}=\left\{\begin{array}{cc} \frac{\lambda }{n+\lambda },s=0 & \\ \frac{1}{2(n+\lambda )},s\neq 0 & \end{array}\right. \end{array}\right.,$$

where $\sqrt{U_{\left(i\right)}}$ denotes the *i*-th column of matrix $\sqrt{U}$, s indicates the s-th row or column, the subscript *m* of the scalar weight *W* denotes the weight associated with the mean, *c* is the covariance with superscript indicating the sigma point index, and λ is a scaling parameter used to reduce overall prediction error, with n+λ applicable for Gaussian distributions.

Step 3: Prediction process.

(13) $$\left\{\begin{array}{l} \chi_{g}^{\left(s\right)}=f\left(\chi_{g-1}^{\left(s\right)}\right),s=1,2,\ldots ,2n\\ {\hat{x}}_{g-1}=\sum_{s=0}^{2n}W_{m}^{s}(\chi_{g}^{\left(s\right)})\\ P_{g}=\sum_{s=0}^{2n}W_{c}^{s}\left(\chi_{g}^{\left(s\right)}-{\hat{x}}_{g-1}\right){\left(\chi_{g}^{\left(s\right)}-{\hat{x}}_{g-1}\right)}^{T}+Q_{g-1} \end{array}\right.,$$

Step 4: Using the one-step-ahead predicted value, perform LU decomposition again to generate a new set of sigma points.

(14) $$\left\{\begin{array}{l} \chi_{g-1}^{\left(0\right)}={\hat{x}}_{g-1}\\ \chi_{g-1}^{\left(s^{\prime }\right)}={\hat{x}}_{g-1}+{\left(\sqrt{n+\lambda }\cdot L\cdot \sqrt{U_{\left(i\right)}}\right)}_{s^{\prime }},s^{\prime }=1,\ldots ,n\\ \chi_{g-1}^{\left(s^{\prime }+n\right)}={\hat{x}}_{g-1}-{\left(\sqrt{n+\lambda }\cdot L\cdot \sqrt{U_{\left(i\right)}}\right)}_{s^{\prime }},s^{\prime }=1,\ldots ,n \end{array}\right.,$$

Step 5: Measurement Update.

(15) $$\left\{\begin{array}{l} {\mathbb{Z}}_{g}=H(\chi_{g}^{\left(s\right)})\\ {\hat{z}}_{g}=\sum_{s=0}^{2n}W_{m}^{s}(Z_{g})\\ P_{{\hat{z}}_{g}}^{\prime }=c_{g}\cdot (\sum_{s=0}^{2n}W_{c}^{s}(Z_{g}-{\hat{z}}_{g}){\left(Z_{g}-{\hat{z}}_{g}\right)}^{T}+R_{g-1})\\ P_{\hat{x}{\hat{z}}_{g}}=\sum_{s=0}^{2n}W_{c}^{s}(\chi_{g}^{\left(s\right)}-{\hat{x}}_{g-1}){\left(Z_{g}-{\hat{z}}_{g}\right)}^{T} \end{array}\right.,$$

Compute the Kalman Gain.

(16) $$K_{g}=P_{\hat{x}{\hat{z}}_{g}}\cdot {\left(P_{{\hat{z}}_{g}}^{\prime }\right)}^{-1},$$

State and Covariance Update.

(17) $${\hat{x}}_{g}={\hat{x}}_{g-1}+K_{g}(Z_{g}-{\hat{z}}_{g}),$$

(18) $$P_{g}=P_{g-1}-K_{g}\cdot P_{{\hat{z}}_{g}}^{\prime }\cdot K_{g}^{T},$$

Step 6: Iterate and repeat the next filtering calculation until completion.

### 3.3. Proposed QLIAO-ELM for Global Fusion

#### 3.3.1. Aquila Optimizer (AO)

The AO simulates four hunting behaviors of the Aquila. According to the number of iterations, these behaviors are divided into the exploration phase and the exploitation phase, where the first 2/3 of iterations correspond to exploration, and the last 1/3 to exploitation. Similar to most swarm intelligence algorithms, individuals in AO possess only one attribute: position. Let the population size of Aquila be *N*, and each eagle’s position in a D-dimensional space be $X=(x^{1},x^{2},\ldots ,x^{D})$. The quality of a given position is evaluated by its fitness function.

(1) Extended Exploration (*X*_1_)

Aquila identifies prey areas and selects the optimal hunting region by soaring at high altitudes and performing vertical dives. During this process, the eagle conducts a broad exploration from high altitudes to determine the search space region of the prey. This behavior is mathematically expressed as shown in (19).

(19) $$X_{1}(t+1)=X_{best}(t)\times \left(1-\frac{t}{T}\right)+(X_{M}(t)-X_{best}(t)*rand),$$

where *X*_1_(*t*+1) denotes the next solution generated at iteration t under extended exploration. *X*_best_(*t*) represents the best solution obtained prior to iteration *t* reflecting the approximate prey location. The term $\left(1-t/T\right)$ regulates the exploration extent based on the iteration count. $X_{M}(t)$ is the mean position of solutions at iteration *t*, calculated as (20). Here, *rand* is a uniform random number in [0, 1], and *t* and *T* represent the current and maximum iteration numbers, respectively.

(20) $$X_{M}(t)=\frac{1}{N}\sum_{i=1}^{N}X_{i}(t),\forall j=1,2,\ldots ,D,$$

(2) Narrowed Exploration $\left(X_{2}\right)$

Upon detecting the prey area, Aquila hovers above the target prey in preparation for descent and attack. This contour flight and short-range gliding attack involves constrained exploration in the selected prey region. The behavior is mathematically formulated as (21).

(21) $$X_{2}(t+1)=X_{best}(t)\times Levy(D)+X_{R}(t)+(y-x)*rand,$$

where $X_{2}(t+1)$ denotes the next solution at iteration t. $X_{R}(t)$ is a randomly selected solution from the population [1, N], at iteration *i*. The variables x and y describe the spiral search trajectory, calculated as (22).

(22) $$\left\{\begin{array}{l} y=r\times cos(\theta )\\ x=r\times sin(\theta )\\ r=r_{1}+U\times D_{1}\\ \theta =-\omega \times D_{1}+\frac{3\times \pi }{2} \end{array}\right.,$$

where $r_{1}$ in [1, 20] defines the search cycle count; U=0.00565 and *ω* = 0.005 are small constants; *D*_1_ is an integer in [1, D], representing the dimension of the search space. The Lévy flight distribution *Levy*(*D*) is computed as (23).

(23) $$Levy(D)=s\times \frac{\mu \times \sigma }{|v{|}^{\frac{1}{\beta }}},$$

where *s* = 0.001 is a constant, and *u,v* ∈ [0,1]. The parameter σ is computed as in (24), here *β* = 1.5.

(24) $$\sigma =\frac{\Gamma (1+\beta )\times \sin(\frac{\pi \beta }{2})}{\Gamma (\frac{1+\beta }{2})\times \beta \times 2^{\left(\frac{\beta -1}{2}\right)}},$$

(3) Extended Exploitation (*X*_3_)

Once the prey area is precisely located and Aquila is ready to attack, it performs a low-flying slow descent attack to observe the prey’s reaction. In this exploitation phase, the eagle approaches the target and initiates the strike. This behavior is expressed in (25).

(25) $$X_{3}(t+1)=\left(X_{best}(t)-X_{M}(t)\right)\times \alpha -rand+((UB-LB)\times rand+LB)\times \delta ,$$

where $X_{3}(t+1)$ denotes the next solution at iteration t under extended exploitation. *X_best_*(*t*) represents the approximate prey location (best solution) at iteration t, and $X_{M}(t)$ is the mean of current solutions, calculated by (20). *rand* ∈ [0,1]. The parameters α and δ control the exploitation adjustment and are set to 0.1. LB and UB represent the lower and upper bounds of the search space.

(4) Narrowed Exploitation $\left(X_{4}\right)$

When approaching the prey, Aquila executes a ground-level capture attack, accounting for the prey’s random movements. This final-stage behavior is formulated in Equation (26).

(26) $$X_{4}(t+1)=QF\times X_{best}(t)-(G_{1}\times X(t)\times rand)-G_{2}\times Levy(D)+rand\times G_{1},$$

where *QF* is a quality function balancing exploration and exploitation, given by (27); $G_{1}$ represents motion patterns used during prey tracking, generated in (28); $G_{2}$ is a decreasing parameter from 2 to 0, representing the flight slope from initial to final position, generated in (29).

(27) $$QF(t)=t^{\frac{2\times rand-1}{{\left(1-T\right)}^{2}}},$$

(28) $$G_{1}=2\times rand-1,$$

(29) $$G_{2}=2\times (1-\frac{t}{T}),$$

where *rand* ∈ [0,1] and t and T denote the current and total iterations, respectively.

In summary, AO introduces four search strategies—extended exploration, narrowed exploration, extended exploitation, and narrowed exploitation—to maintain a balance between exploration and exploitation. Through iterative updates, each solution converges toward the optimal solution according to the best solution obtained during the optimization process. A greedy selection mechanism ensures that each new position is superior to the previous one in both phases.

#### 3.3.2. Improved AO

Although the AO algorithm exhibits fast convergence and strong exploration capabilities, it still suffers from slow convergence rates and susceptibility to local optima during the optimization process. By analyzing the mathematical model of the original AO, it can be observed that among the four position update formulas, (19) and (26) converge to zero, while (25) converges to a constant. This characteristic significantly degrades the algorithm’s performance when addressing complex engineering problems. To address these limitations, this study proposes a Q-learning-driven Improved AO (QLIAO), with the specific enhancements described below.

(1) Opposition-Based Learning Initialization

To explore unknown optimal solutions in the search space, Tizhoosh [39] proposed the opposition-based learning (OBL) model based on estimation and counter-estimation. In this work, OBL is employed to increase the diversity of the initial population, enabling thorough exploration in the early iterations of the AO algorithm. The relevant definitions are as follows:

First, the initial population *pop*_1_ is generated randomly according to (30). Then, the opposite population *pop*_2_ is generated using opposition-based learning as in (31). Finally, the fitness of individuals in both populations is calculated, sorted in ascending order, and the top N individuals are retained as the final initial population

(30) X=rand×(UB−LB)+LB,

(31) $$X^{\prime }=UB+LB-X,$$

where *rand* is a random value between 0 and 1, and LB and UB represent the lower and upper bounds of the given problem, respectively.

(2) Spiral Encircling Attack Mechanism

The spiral encircling operator, previously applied in the Moth-Flame Optimization (MFO) [40] and Whale Optimization Algorithm (WOA) [18], enables solutions to spiral around a flame or prey while approaching it. Replacing the original AO’s expansion behavior (*X*_3_) with this strategy effectively enhances the algorithm’s ability to escape local optima. The updated position formula for the modified expansion behavior (*X*_3_) is expressed as:

(32) $$X_{3^{\prime }}(t+1)=X_{best}(t)+|X_{best}(t)-X(t)|\cdot e^{bl}\cdot \cos(2\pi l),$$

(3) Q-Learning-Based Neighborhood Disturbance Operator

Q-Learning is a classical algorithm in reinforcement learning, whose core idea is to store the Q-values corresponding to state-action pairs in a Q-table. An agent selects the optimal action based on Q-values to maximize cumulative rewards. Essentially, Q-Learning is a Markov Decision Process (MDP)-based reinforcement learning method, where a discretized matrix serves as the action-value function. Its basic components include agents, environment, states, actions, and rewards. Specifically, after an agent performs an action, the environment provides feedback in the form of a reward; the agent then selects and executes a new action based on the updated state and received reward.

The iteration process of AO can be modeled as a Markov process. The eagle represents the agent, the state space consists of 0 and 1, the action space is defined by a policy pool, and the population serves as the environment. *Q_t_*(*s,π*) denotes the expected reward obtained by executing policy π in state *s*. The algorithm constructs a Q-table to store Q-values for state-policy pairs, and the strategy yielding the maximum expected reward is selected. The Q-values in the table are updated using the temporal difference method, as given by (33):

(33) $$\left.Q_{t}(s,\pi )=Q_{t-1}(s,\pi )+\rho *(R+\gamma *\max\left(Q_{t-1}(s,\pi )\right)-Q_{t-1}(s,\pi )\right),$$

(34) $$R=\left\{\begin{array}{l} \omega \times t\times |\log(Best_{t+1})-\log(Best_{t})|,Best_{t+1}>Best_{t}\\ -0.5,Best_{t+1}=Best_{t} \end{array}\right.,$$

where ρ and γ denote the learning rate and discount factor, respectively; *R* is the reward function computed by Equation (34); ω is an adjustment factor; and *Best_t_* represents the best fitness at iteration t.

The strategy corresponding to the maximum Q-value is selected to update the population. The strategy pool contains the following two strategies:

Strategy 1: Heterogeneous t-Distribution Perturbation $\left(X_{Q1}\right)$

A heterogeneous degree t-distribution perturbation operator is introduced to randomly perturb population individuals, enhancing the algorithm’s diversity and exploration capability. The heterogeneous t-distribution perturbation is formulated as:

(35) $$X_{Q1}(t+1)=\frac{t}{iter\_max}\cdot X_{trnd(10)}^{t}+(1-\frac{t}{iter\_max})\cdot X_{trnd(2)}^{t},$$

where *trnd*(10) and *trnd*(2) denote t-distributions with 10 and 2 degrees of freedom, respectively.

After perturbation, the Metropolis criterion is used to accept inferior solutions with a certain probability *P*, preventing premature convergence to local optima. *P* is computed as:

(36) $$P=\exp(-\frac{\Delta F}{T_{e}}),$$

where *T_e_* represents the current temperature, and Δ*F* is the fitness difference between the new solution and the current solution. The initial temperature is set to 100, and the cooling coefficient is 0.9.

For minimization problems, if Δ*F* ≤ 0, the new solution is accepted, i.e., $X_{Q1}(t+1)=X_{Q1}(new)$; otherwise, the new solution is accepted with probability *P*. A random number *rand* ∈ [0,1] is generated, and if rand<P, the new solution is accepted; otherwise, the current solution is retained, i.e., *X*_*Q*1_(*t*+1) = *X*_*Q*1_(*t*).

Strategy 2: Differential Evolution $\left(X_{Q2}\right)$

In the differential evolution (DE) algorithm, each individual encodes a candidate solution to the problem. During each iteration, a mutation operation is first applied: a base individual is selected, and differences from one or more other individuals are scaled and added to the base to generate a new individual. A crossover operation then combines the new individual with the parent individual, followed by a selection operation that retains the better individual for the next generation. After all iterations, the best individual in the population is selected as the solution [41]

The mutation operation is defined as:

(37) $$X_{Q2^{\prime }}(t+1)=X_{r1}(t)+Q(X_{r2}(t)-X_{r3}(t)),$$

where $X_{r1}$, $X_{r2}$, and $X_{r3}$ denote three randomly selected individuals, and *Q* is the scaling factor, set to 0.5.

The crossover operation is expressed as:

(38) $$X_{Q2^{\prime \prime }}(t+1)=\left\{\begin{array}{c} X_{Q2^{\prime }}(t+1),\;if\;rand(0,1)\leq CR\\ X(t),\;\;\;\;\;otherwise\; \end{array}\right.,$$

where *CR* is the crossover probability.

The selection operation is given by:

(39) $$X_{Q2}(t+1)=\left\{\begin{array}{c} X_{Q2^{\prime \prime }}(t+1),\;if\;F(X_{Q2^{\prime \prime }}(t+1)\leq F(X(t)))\\ X(t),\;\;\;\;\;\;\;otherwise\; \end{array}\right.,$$

In this study, the improved strategy consists of three core components: the reward matrix, the Q-matrix, and the Bellman equation. The reward matrix represents rewards and penalties associated with the agent’s actions or states, typically in a two-dimensional matrix where each state-action pair receives either a positive reward (+1) or a negative penalty (−1). The Q-matrix reflects the accumulated experience of the agent during previous iterations, where each value represents the expected outcome of selecting a specific action in a given state.

At the initial stage, the agent has no prior knowledge or experience of the environment, so the Q-matrix is initialized as a zero matrix. As the agent explores the environment over multiple iterations and updates the Q-matrix using the Bellman equation, it gradually accumulates experience, improving its decision-making process.

Specifically, when the Q-value of the self-cognition perturbation operator is maximal in a given state, the agent adjusts its position according to the self-cognition perturbation equation. Other perturbation strategies follow the same procedure. During this process, rewards are assigned by comparing the current fitness with the updated fitness: if the new fitness is better, the agent receives a positive reward (+1); otherwise, it receives a negative penalty (−1).

**Figure 3:**
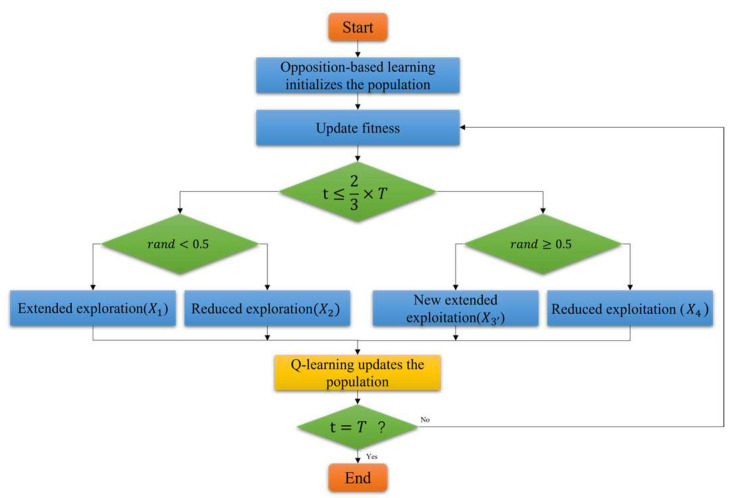
Flowchart of the QLIAO algorithm.

The flowchart of the improved QLIAO is illustrated in Figure 3.

Combining the AO algorithm with Q-Learning offers the following advantages: (1) Q-Learning can learn search directions that are more likely to lead to optimal solutions during the search process and transfer this knowledge to the AO algorithm, thereby guiding subsequent searches. (2) By integrating Q-Learning, the AO algorithm can intelligently select search strategies and parameters, enabling a more comprehensive exploration of the search space. (3) The combination of AO and Q-Learning enhances the algorithm’s adaptability and generalization capability. While a single metaheuristic algorithm typically relies on parameter tuning to address specific problems, it may require re-tuning or alternative search strategies when the problem changes. In contrast, integrating Q-Learning allows the algorithm to adapt to different problems through learning, thereby achieving improved adaptability and generalization.

Therefore, in this study, the QLIAO algorithm will be employed to optimize the neural network model.

#### 3.3.3. QLIAO-ELM for Global Data Fusion

The Extreme Learning Machine (ELM) is an innovative feedforward neural network learning method that demonstrates remarkable efficiency in the field of machine learning due to its unique design. The core parameters of ELM are the inter-layer connection weights and hidden layer biases, which can be randomly initialized and remain fixed during training, thereby simplifying the network architecture and avoiding the iterative parameter updates required by traditional models. During training, since these parameters do not require frequent optimization, only the output weights need to be calculated. This significantly reduces inter-layer computational cost while maintaining learning accuracy, greatly enhancing training speed and addressing the time-consuming issue of conventional feedforward neural networks. In addition, ELM exhibits excellent generalization capability, making it suitable for regression tasks and effective in building stable models from small-sample datasets, thus providing an efficient solution for small-sample learning [42,43,44,45].

In this study, ELM is selected as the global data fusion model for multi-anemometer measurements, primarily due to its strong alignment with the requirements of the application scenario: (1) multi-anemometer systems require real-time processing of multisource parallel data, and the fast learning and computation ability of ELM prevents data accumulation delays, thereby meeting real-time fusion demands; (2) heterogeneous sensors are prone to environmental interference and data deviation, and the robust generalization capability of ELM effectively accommodates data variability, reducing the impact of noise on the fusion results; (3) the property of not requiring repeated parameter tuning reduces system calibration cost, thereby improving engineering applicability.

The original activation function of ELM is Softplus. However, because multi-anemometer data are often subject to small fluctuations caused by noise, the Softplus function—being highly sensitive to input changes—may amplify such noise. In contrast, the Sigmoid function saturates at larger absolute input values (where the output change approaches zero), thereby naturally suppressing noise effects and improving the stability of ELM-based fusion. Hence, Sigmoid is adopted as the activation function in this study, expressed as in (40):

(40) $$f(x)=\ln(1+e^{x}),$$

Since the initial weights and biases of ELM are randomly generated, the stability of the model may be compromised. To address this issue, the proposed QLIAO algorithm is employed to optimize these parameters, ensuring that the ELM yields more stable and accurate fusion results during global data integration.

In the parameter optimization process, the mean squared error (MSE) of the training set is used as the fitness function, with minimization of this metric guiding the search for optimal parameters. The fitness function is defined as (41).

(41) fitness=argmin(MSE),

A smaller training set MSE indicates a higher consistency between model predictions and the original data, thereby signifying that the weights and biases optimized by QLIAO are superior. Once the optimal initial parameters are obtained, the ELM network is trained accordingly and subsequently tested on the validation dataset.

The specific procedure of ELM optimization using QLIAO is summarized as follows:

Step 1: Input the training dataset and specify the number of hidden neurons in the ELM.

Step 2: Employ the QLIAO algorithm to optimize and obtain the best input weights and hidden biases.

Step 3: Compute the hidden layer output matrix with the optimized parameters, derive its Moore–Penrose pseudoinverse, and calculate the output layer weights.

Step 4: Integrate the above computations to generate the final fused wind speed estimation.

In the QLIAO-ELM global fusion model, the number of hidden layer neurons in ELM is a key parameter affecting the model’s fitting ability and generalization capability. The determination is based on the optimal method formula, $q=\sqrt{m+n}+a$, where q is the number of hidden layer neurons, m is the number of input layer neurons, n is the number of output layer neurons, and a is a constant less than 10. In this paper, the input to the global fusion stage consists of 2 sets of data from the local fusion output, so m=2. The output is a unidimensional wind speed value, so n=1; thus, q∈[3,12].

Next, the trial-and-error method is used to test the impact of different q values within the theoretical range on the performance of the ELM fusion. Using the sensor data samples from the local fusion stage, the first 420 sets of data are used as training samples, the middle 90 sets are used as the validation samples, and the remaining 90 sets are used as test samples. The RMSE of the global fusion model is taken as the core evaluation metric. The final result shows that when the number of neurons is 8, the root mean square error (RMSE) is minimized. Therefore, in this paper, the number of hidden layer neurons in ELM is set to 8.

## 4. Time Complexity Analysis

In local fusion, the complexity of the traditional UKF is $O\left(n^{3}\right)$, where n is the dimension of the state space. FLR-UKF introduces three core operations based on the traditional UKF: (1) Fuzzy logic adaptive weighting mechanism. This operation involves calculating the normalized innovation sequence and covariance ratio, performing fuzzy rule inference, and centroid defuzzification, with a complexity of $O\left(m\right)$, where m is the number of rules in the fuzzy logic system. (2) Robustness factor correction. This operation only requires scalar multiplication on the innovation covariance, with a complexity of $O\left(1\right)$. (3) LU decomposition. This operation decomposes the n×n  covariance matrix, with a complexity of *O*(*n*^3^). Therefore, the total computational complexity of FLR-UKF remains *O*(*n*^3^), which is the same as the traditional UKF, and the performance improvement does not lead to a significant increase in computational cost.

In global fusion, QLIAO-ELM is used to perform the global fusion. The core complexity of ELM is $O\left(L\times M+L^{2}\right)$, where L is the number of hidden layer neurons and M is the number of samples. The complexity of the QLIAO optimization phase is $O\left(N\times T\times L\right)$, where N is the population size and T is the number of iterations. The core improvements (oppositional learning initialization, spiral wrapping attack mechanism, and Q-learning neighborhood disturbance operator) do not increase the order of complexity, and still maintain the same order of complexity as traditional intelligent optimization algorithms such as AO, PSO, and JADE. Moreover, the adaptive strategy of Q-learning can accelerate convergence, thereby reducing the actual iteration cost.

In summary, the hierarchical fusion strategy proposed in this paper does not lead to an increase in time complexity. FLR-UKF provides high-quality input data for global fusion through precise denoising and outlier suppression, reducing redundant searches in subsequent optimization. The Q-learning adaptive strategy and oppositional learning initialization in QLIAO accelerate the convergence speed, reducing ineffective iterations. Combined with the fast computation characteristics of ELM, this ultimately results in a reduction in the actual runtime of the overall fusion process.

## 5. Simulation and Discussion

### 5.1. Performance Evaluation of the QLIAO Algorithm

To evaluate the overall performance of the proposed QLIAO algorithm in terms of solution accuracy and convergence speed, comparative experiments were conducted on the CEC2019 benchmark function set [43], against AO, PSO [44], and JADE [45]. In the CEC2019 test suite, functions F1–F3 have dimensions of 9, 16, and 18, respectively, while functions F4–F10 are 10-dimensional minimization problems. As most functions in CEC2019 are multimodal, the benchmark is highly challenging.

The experimental parameters were configured as follows: the population size was set to 100, and the maximum number of iterations was fixed at 500. The best solutions, the mean of the best solutions, and the standard deviations obtained by the four algorithms are summarized in Table 1. To eliminate the influence of randomness, each algorithm was independently executed 30 times, ensuring the reliability of the test results.

Table 1 presents the optimization performance of the four algorithms on the CEC2019 benchmark functions, with the comparative algorithm values highlighted in bold. The proposed QLIAO algorithm demonstrates consistently superior overall performance compared to the other three algorithms.

**Figure 4:**
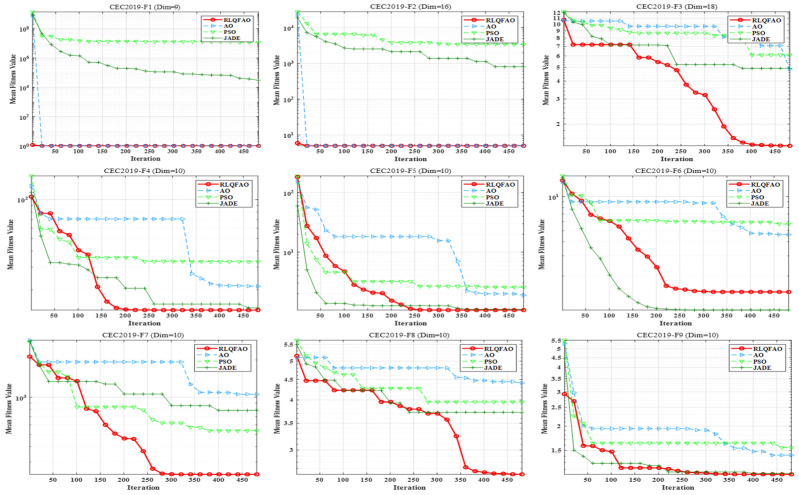
Convergence curves.

To visually compare the optimization performance, Figure 4 illustrates the convergence curves of the four algorithms on the CEC2019 functions [46]. The results indicate that, relative to AO, PSO [47], and JADE [48], QLIAO achieves the best performance in terms of convergence accuracy, convergence speed, and stability. On more than eight test functions, QLIAO exhibits stronger optimization capability and faster convergence. This improvement is attributed to Q-Learning, which enables the algorithm to learn search directions that are more likely to lead to optimal solutions and transfer this knowledge to guide subsequent AO searches. Moreover, the integration of Q-Learning allows AO to intelligently select search strategies and parameters, thereby enabling a more comprehensive exploration of the search space.

In summary, the QLIAO algorithm demonstrates significant advantages in both convergence speed and optimization performance.

### 5.2. Experimental Data

The real wind speed reference data used in this experiment is sourced from a 30 m wind measurement tower at a grid-connected wind farm in Gansu Province, China. The tower is equipped with high-precision wind speed sensors, and the data collection period was from 1 January to 7 January 2023. After excluding invalid data caused by equipment malfunctions and extreme weather conditions (such as sandstorms), 600 sets of continuous and stable wind speed data were selected as the baseline for the subsequent artificially generated sensor data.

A dataset comprising six ordinary sensors was generated by superimposing nonlinear noise or disturbances onto these measurements. Nonlinear interference is a core source of error commonly found in standard wind speed sensors operating in complex environments. It is not a subjective fabrication, but rather a true reflection of the output deviations caused by environmental factors and the inherent limitations of the sensor’s performance in real-world applications. This method of sensor data generation uses high-precision wind speed data from an actual wind farm as the baseline, adding only nonlinear noise or interference. This approach avoids distorting the physical meaning of the data, while enabling the precise construction of experimental scenarios with multiple sensors and interference sources. It provides representative input data for the subsequent performance validation of the FLR-UKF local denoising and QLIAO-ELM global fusion algorithms, ensuring that the algorithms’ effectiveness and applicability in real interference scenarios can be thoroughly tested.

### 5.3. Analysis of Local Fusion Results

To validate the effectiveness of the algorithm, 600 high-precision wind speed measurements collected from a wind farm in China were used as the true wind speed. A dataset comprising six ordinary sensors was generated by superimposing nonlinear noise or disturbances onto these measurements. Local fusion was tested on the first 200 samples. Specifically, the data from the six sensors were randomly divided into two groups, and the FLR-UKF was applied for local fusion. Simulations were conducted using MATLAB R2021b. The fusion performance was evaluated using four metrics: RMSE, MAE, MRE, and *R*^2^.

**Figure 5:**
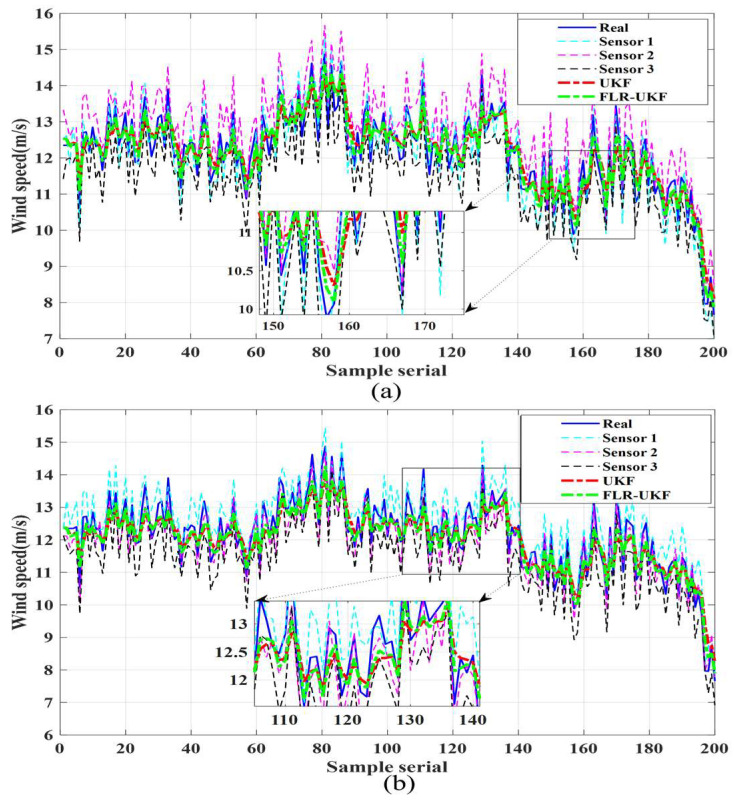
Comparison of local fusion results. (**a**) Local fusion of the first group of underlying sensor data. (**b**) Local fusion of the second group of underlying sensor data.

Figure 5 illustrates the variations in the two groups of true wind speeds, the raw measurements from the underlying sensors, and the fusion results obtained by UKF and FLR-UKF along the sample sequence. Overall, although UKF can fit the true wind speed to some extent, noticeable deviations are observed in certain local sample intervals. In contrast, the FLR-UKF trajectories align more closely with the true values. Particularly in regions with abrupt wind speed changes (e.g., sample sequence 150–170 in Figure 5a), FLR-UKF corrects abnormal fluctuations in the underlying sensor data more accurately, demonstrating superior nonlinear fitting and tracking capability.

This result directly reflects the adaptability of the fuzzy logic adaptive weighting mechanism in the FLR-UKF to non-stationary noise, as well as the suppressive effect of the robustness factor on outliers. When sensor data undergoes sudden changes due to interference from complex environmental factors, FLR-UKF is able to more accurately capture the true wind speed characteristics, thereby avoiding trajectory drift caused by the fixed noise covariance assumption in traditional UKF.

**Figure 6:**
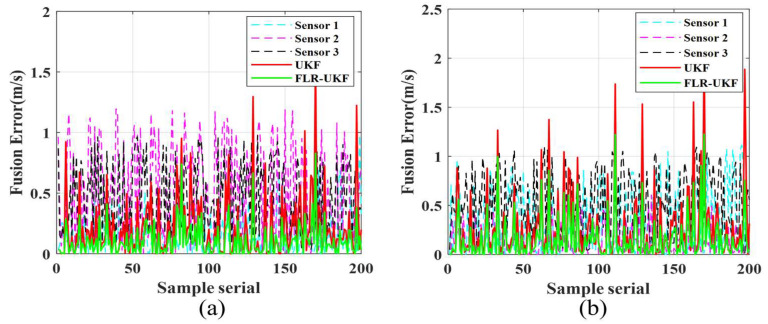
Comparison of fusion errors using different algorithms. (**a**) Local fusion errors of the first group. (**b**) Local fusion errors of the second group.

Figure 6a,b depict the fusion errors of the two groups of underlying sensor data using different algorithms along the sample sequence. The horizontal axis represents the sample sequence, while the vertical axis denotes the error magnitude. The three sensors’ data reflect the quality variations in the original sensor measurements. During the fusion process of both groups, the UKF exhibits larger error amplitudes and frequent fluctuations, indicating limited capability in noise suppression and error correction. In contrast, FLR-UKF shows significantly reduced error magnitudes with smoother fluctuations, demonstrating the advantage of the improved algorithm in error control and its ability to provide more stable fusion results.

This result validates that FLR-UKF ensures covariance positive definiteness through the robustness factor correction and LU decomposition, effectively reducing the interference of outliers on the fusion results and improving the stability of data output.

**Figure 7:**
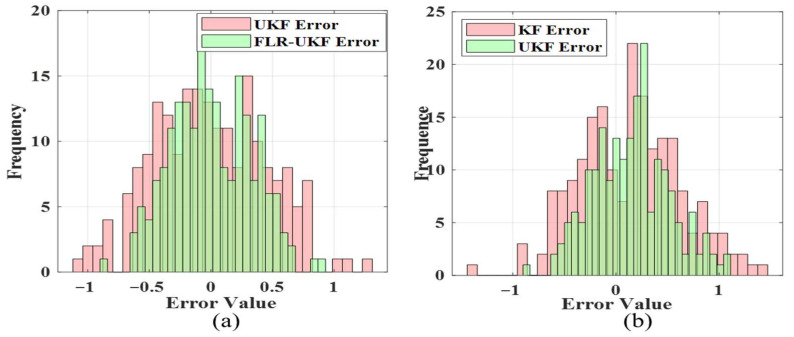
Statistical distribution of fusion errors. (**a**) Error distribution of the first group’s local fusion. (**b**) Error distribution of the second group’s local fusion.

Figure 7a,b present the error distributions of UKF and FLR–UKF, with error values on the horizontal axis and frequency on the vertical axis. It can be observed that the UKF errors are widely distributed, covering the range from −1 to 1, with a scattered distribution on both sides of zero, indicating high randomness and poor stability. In comparison, FLR-UKF errors are concentrated within the range of −0.5 to 0.5, with higher peaks and a more compact distribution, demonstrating that the improved algorithm produces errors closer to zero. The higher concentration and consistency of the error distribution indicate improved reliability of the fusion results.

Table 2 presents a comparison of quantitative performance metrics. RMSE reflects the overall dispersion of errors. For the first group, UKF1 achieves 0.4566, while FLR-UKF1 decreases to 0.3259, representing a 28.6% reduction. For the second group, UKF2 is 0.5105, and FLR-UKF2 drops to 0.3957, a reduction of 26.46%. This indicates that the improved algorithm provides better overall error control.

MAE measures the average magnitude of errors. UKF1 has an MAE of 0.3759, which is reduced to 0.2698 by FLR-UKF1 (approximately 28.2% decrease). For the second group, UKF2 has an MAE of 0.4141, while FLR-UKF2 reduces it to 0.3176 (about 23.3% decrease), demonstrating the effectiveness of FLR-UKF in suppressing deviations in the fused results.

MRE represents the relative error with respect to the true values. UKF1 records 0.0314, and FLR-UKF1 improves to 0.0223. UKF2 is 0.0342, and FLR-UKF2 reduces it to 0.0259, indicating that FLR-UKF yields smaller relative deviations and better percentage error control.

The coefficient of determination *R*^2^ reflects the model’s goodness of fit, with values closer to 1 indicating better fitting. UKF1 achieves 0.8428, while FLR-UKF1 rises to 0.9199. UKF2 is 0.8035, with FLR-UKF2 increasing to 0.8820, showing that FLR-UKF can better capture the variability of the true wind speed and significantly enhance model fitting.

Overall, through multidimensional visualization and quantitative metric analysis, FLR-UKF demonstrates significant advantages in wind speed data fusion. Its fusion trajectories closely follow the true values, effectively correcting abnormal sensor fluctuations. In terms of error control, RMSE, MAE, MRE, and *R*^2^ metrics all outperform the conventional UKF, confirming the effectiveness and superiority of FLR-UKF in multi-sensor nonlinear data fusion scenarios. This makes it a preferable choice for sensor data fusion in complex environments.

### 5.4. Analysis of Global Fusion Results

**Figure 8:**
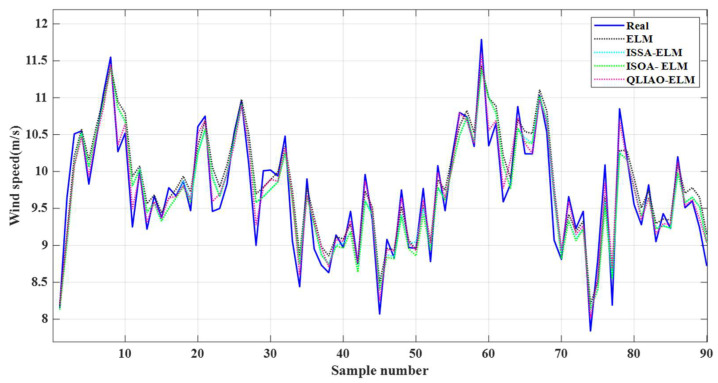
Comparison of global fusion results.

The QLIAO–ELM model was applied for global fusion and compared with ELM, ISSA-ELM [29], and ISOA-ELM [49]. Figure 8 presents the time-series comparison of the fused wind speed. Overall, the QLIAO-ELM curve exhibits a closer fit to the true wind speed. In the locally enlarged segment of sample numbers 35–55, QLIAO-ELM predictions track the true values more tightly, whereas the predictions of ELM and the other models show noticeable deviations from the true curve. This preliminary observation suggests that QLIAO-ELM has superior temporal tracking capability in wind speed fusion.

Table 3 summarizes the evaluation metrics of the four fusion models. For ELM, RMSE is 0.3032, MAE is 0.2474, MRE is 0.0253, and *R*^2^ is 0.8496. The ELM models optimized with ISSA and ISOA show reduced error metrics and improved *R*^2^. In comparison, QLIAO-ELM achieves an RMSE of 0.2511, MAE of 0.2149, MRE of 0.0228, and *R*^2^ of 0.8759, representing the lowest errors and the highest *R*^2^ among all models. These results demonstrate the significant optimization effect of QLIAO on ELM, indicating that QLIAO-ELM outperforms the other models in both wind speed prediction accuracy and model fitting quality.

Neural networks can achieve better results in data fusion, but their computational complexity and running time are relatively high, which may affect the real-time performance of the fusion system. Therefore, Table 4 presents the computation times for fusing 600 sets of wind speed data using four different neural networks. Based on the running times in Table 4, it can be seen that the ELM algorithm has the fastest speed and is suitable for real-time fusion. The proposed QLIAO-ELM algorithm takes 2.378 s to fuse 600 sets of data, which is faster than both the ISSA-ELM and ISOA-ELM algorithms. The advantage of the proposed QLIAO-ELM algorithm in fusion speed is attributed to the simplicity of the ELM and the faster convergence speed of the improved QLIAO algorithm.

## 6. Conclusions

This study addresses challenges in wind speed sensor data fusion at meteorological towers, including noise interference, nonlinear system errors, and insufficient real-time performance. A hierarchical data fusion algorithm is proposed, combining local data fusion with global decision-level fusion to effectively enhance the accuracy and reliability of wind speed measurements. The results indicate that the FLR-UKF algorithm exhibits excellent noise reduction and outlier suppression capabilities during the local fusion stage, while the QLIAO-ELM model demonstrates superior performance over comparative models in the global decision-level fusion stage, confirming its effectiveness in handling multi-sensor wind speed data.

Although the proposed hierarchical fusion method achieves promising results in meteorological tower wind speed monitoring, there remains room for further extension and optimization. Future research will focus on two key aspects: First, exploring asynchronous fusion mechanisms for multi-rate sensor data. The current algorithm assumes uniform sensor sampling frequencies, whereas in practice, sensors at different heights may have different sampling rates, necessitating spatiotemporal alignment strategies for asynchronous data scenarios. Second, enhancing algorithm adaptability under extreme conditions. For issues such as data loss or abrupt changes under strong winds, dust storms, and other harsh environments, integrating federated learning and attention mechanisms could improve the model’s fault tolerance and dynamic response to incomplete data.

## Figures and Tables

**Table 1:** Results of the best solution, mean best solution, and standard deviation obtained by the four algorithms.

No.	Metrics	RLQFAO	AO	PSO	JADE
F1	min	**1**	1	5,264,481	10,367.33
	std	**0**	1.35 × 10^−11^	3,337,546	15,203.07
	mean	**1**	1	9,795,371	25,589.8
F2	min	**5**	5	2825.251	810.1548
	std	**0**	0	662.7351	171.1976
	mean	**5**	5	3860.759	1064.18
F3	min	**1.409275**	3.498918	6.092859	4.901912
	std	**0.526567**	0.602424	1.188343	0.684858
	mean	**1.981562**	4.445345	7.971577	5.536331
F4	min	**11.18122**	19.00174	27.38198	13.93446
	std	**1.907375**	8.644842	7.053731	8.316466
	mean	**14.5196**	26.4566	35.77686	20.23583
F5	min	**1.036956**	1.535422	2.223836	1.056569
	std	**0.034855**	0.178872	0.228386	0.042565
	mean	**1.098233**	1.740111	2.49403	1.134598
F6	min	1.44366	2.130894	3.355768	1
	std	1.579856	1.718993	1.552666	3.5 × 10^−5^
	mean	2.808948	4.799874	5.198204	1.000014
F7	min	**238.5789**	680.367	536.6835	782.645
	std	**124.3266**	229.5847	432.6898	159.5378
	mean	**584.7184**	1116.707	980.4762	1028.304
F8	min	**2.60863**	3.363507	3.766207	3.205121
	std	**0.295815**	0.460476	0.131893	0.299592
	mean	**3.261958**	4.290651	3.950581	3.517968
F9	min	**1.101835**	1.247775	1.392617	1.130638
	std	**0.017688**	0.07402	0.141671	0.042733
	mean	**1.128022**	1.362128	1.54351	1.174628
F10	min	21.00799	3.413	21.14169	14.74046
	std	0.100223	8.76344	0.122043	2.607685
	mean	21.1629	15.55236	21.36609	19.41916

**Table 2:** Comparison of quantitative performance metrics.

Algorithm	Evaluation Indicators			
	RMSE (m/s)	MAE (m/s)	MRE (m/s)	*R*^2^ (%)
UKF1	0.4566	0.3759	0.0314	0.8428
UKF2	0.5105	0.4141	0.0342	0.8035
FLR-UKF1	0.3259	0.2698	0.0223	0.9199
FLR-UKF2	0.3957	0.3176	0.0259	0.8820

**Table 3:** Evaluation metrics of the four fusion models.

Algorithm	Evaluation Indicators			
	RMSE (m/s)	MAE (m/s)	MRE (m/s)	*R*^2^ (%)
ELM	0.3032	0.2474	0.0253	0.8496
ISSA-ELM	0.2809	0.2236	0.0232	0.8709
ISOA-ELM	0.2795	0.2246	0.0233	0.8722
QLIAO-ELM	0.2511	0.2149	0.0228	0.8759

**Table 4:** Running Times of Several Neural Network Fusions.

Algorithm	ELM	ISSA-ELM	ISOA-ELM	QLIAO-ELM
Time/s	2.131	2.497	2.644	2.378

## Data Availability

Data are contained within the article.

## Abbreviations

The following abbreviations are used in this manuscript:

UKF	Unscented Kalman filter
AO	Aquila Optimizer
ELM	Extreme Learning Machine
FLR-UKF	Unscented Kalman filter enhanced with fuzzy logic and a robustness factor
QLIAO-ELM	Extreme learning machine neural network optimized by a Q-learning–improved Aquila Optimizer
MDF	Multi sensor Data Fusion
WSN	Wireless Sensor Network
FC	Fusion Center
HAR	Human Activity Recognition
ICI	Inverse Covariance Intersection
PDCI	Parallel Deep predictive model
ISOA	Improved Seagull Optimization Algorithm
